# Effect of an electronic reminder of follow-up screening after pregnancy complicated by gestational diabetes mellitus: a randomized controlled trial

**DOI:** 10.1186/s12889-023-15060-9

**Published:** 2023-01-23

**Authors:** Jane Hyldgaard Nielsen, Kirsten Fonager, Jette Kolding Kristensen, Charlotte Overgaard

**Affiliations:** 1 Department of Midwifery, University College of Northern Denmark, Aalborg Øst, Denmark; 2 Public Health and Epidemiology Group, Department of Health Science and Technology, Aalborg University, Aalborg, Denmark; 3 Research Center for Health and Applied Technology, University College of Northern Denmark, Aalborg, Denmark; 4 DECIPH’er, Cardiff School of Social Sciences, Cardiff University, Cardiff, Wales; 5 Department of Social Medicine, Aalborg University Hospital, Aalborg, Denmark; 6 Department of Clinical Medicine, Aalborg University, Aalborg, Denmark; 7 Centre for General Practice, Aalborg University, Aalborg, Denmark; 8 Unit of Health Promotion, Department of Public Health, University of Southern Denmark, Esbjerg, Denmark

**Keywords:** Gestational diabetes mellitus, Type 2 diabetes, Follow-up screening, Reminder, Health prevention, Health research

## Abstract

**Aim:**

To determine the effectiveness of despatching an electronic reminder of participation in screening for gestational diabetes. The reminder was sent to the women 1–8 years after delivery.

**Methods:**

A registry-based, randomized controlled trial in the North Denmark Region among women with gestational diabetes. Randomization was made, which included seven groups stratified by the child’s birth year (2012–2018). The intervention group received standard care supplemented by an electronic reminder through a secure nationwide email system (*n* = 731), while the control group received only standard care (*n* = 732). The primary outcome was based on blood testing for diabetes (OGTT, HbA1c or fasting P-glucose).

**Results:**

A total of 471 (32.1%) women participated in screening. The primary outcome was experienced by 257 women (35.1%) in the intervention group and 214 women (29.2%) in the control group. The effect of the reminder seemed to increase with recipient’s age, non-western origin, urban dwelling, and multiparity. Of those who participated in follow-up screening, 56 (3.8%) were diagnosed with type 2 diabetes.

**Conclusion:**

Electronic reminders, based on the principles of informed choice and patient-centred care, to women have been shown to support life-long participation in follow-up screening. Attempts to further stimulation of coverage could however be considered.

**Trail registration:**

ISRCTN registry (22/04/2022, ISRCTN23558707).

## Introduction

The prevalence of gestational diabetes (GDM) varies regionally, thus affecting approximately 7–8% of pregnancies in Europe [1] and 3–4% of pregnancies in Denmark [2]. These variations can be explained by different screening strategies, diagnostic criteria and population groups [1]. Nonetheless, in addition to increasing the risk of pregnancy complications, women with pregnancy complicated by GDM are at an approximately eightfold higher lifetime risk of type 2 diabetes mellitus after pregnancy [3], as well as an increased risk of cardiovascular diseases [4]. Guidelines therefore recommend follow-up screening 2–3 month after birth and every 12 months, or at least every three years [5]. The low uptake of postpartum screening is, however, a challenge shared by many countries [6, 7]. With approximately 17% of women participating in screening 4–6 years after birth [6], Denmark has seen a drop in the uptake, which is problematic as attendance and receiving a diagnosis of diabetes are positively associated [6]. The untapped potential for early diabetes detection recently led the International Diabetes Federation (IDF) to call for further research into the field [4].

Although reminder interventions have been found effective in targeting some of the many documented barriers to follow-up screening after birth, the effect varies strongly across settings [8]. Our recently published review affirmed the influence of several contextual factors [8] on institutional, community and policy levels. These factors include the need for continuity and collaboration on care across healthcare sectors, standardization of care, logistics, and compensation of the women’s expenses, challenging work obligations and time spent on screening [8].

As previous studies of the effect of reminder interventions have followed women for only 12 months after birth, the available data concern the short-term effects of screening [8–10]. As we know that women with previous GDM remain at high risk of diabetes for at least 15 years after birth [11], the declining participation rates [6] are worrying and call for exploration of the long-term effect of reminder interventions.

The aim of this study was to determine the effectiveness of an electronic reminder intervention targeting women whose pregnancy was complicated by GDM with regard to increasing participation in follow-up screening in general practice clinics. All women in the cohort who had delivered between 2012 and 2018 were eligible.

## Materials and methods

### Study design and participants

#### Design and setting

This study was designed as a two-armed, single-blinded randomized controlled trial. The setting was the North Denmark Region, with approximately 0.6 million inhabitants [12]. Denmark offers all citizens universal healthcare free of charge [12], including follow-up screening for women with previous GDM [13]. It is however up to the women themselves to remember to make use of this offer. General practitioners (GPs) play a key role in all aspects of primary healthcare [12], including follow-up care. The identification of study population, obtaining information on baseline characteristics, despatching reminders and assessing outcomes were enabled by the Danish civil registration system. Holding a permanent and unique number (CPR number) for all residents, the system enables the linkage of individual data across multiple nation-wide registers while ensuring total anonymity [12].

#### Participants

##### Inclusion and exclusion criteria

Women who had given birth between 2012 and 2018 and were diagnosed with GDM were eligible for inclusion. Diagnostic test during pregnancy consists of an oral glucose tolerance test (OGTT) with diagnostic criteria of a 2-h blood glucose (≥ 9.0 mmol/l) [13]. In attempts to strengthen the credibility of GDM diagnosis, women also diagnosed with diabetes (e.g., type 1 or type 2 diabetes mellitus) more than 40 weeks prior to birth were excluded. We also excluded women who had already been diagnosed with diabetes during/after pregnancy prior to the intervention, women who no longer lived in the region or who had died. If a woman was registered with more than one GDM-affected pregnancy, inclusion was based on the youngest live-born child.

##### Identification and data sources

Women were identified via the National Patient Register, which is based on all hospital admissions in Denmark, contains personal information on patients, including CPR number, home municipality, age, parity (primipara or multipara), BMI and death [14]. The Danish Health Service's classification system, reporting on hospital admissions to the National Patient Register, is based on WHO’s International Statistical Classification of Diseases and Related Health Problems (ICD-10) [15], and enabled this identification (e.g., birth (ICD10:D080-D084), GDM-diagnosis (ICD10: D024) and other diabetes diagnosis (ICD10:DE10-14)). Information on ethnicity and employment status was retrieved from the Danish Employment's database (DREAM database) [14].

##### Sample size

The sample size was determined on the basis of a risk difference calculation. Based on participation rates in the region and a reminder intervention achieving an effect above 10% points [6, 16, 17], it was estimated that 388 women per arm were required to detect at least a 10%-point improvement in participation and increase test attendance from 50 to 60%, with 80% power, α of 0.05 and expectation of no loss to follow-up.

### Study intervention

In the setting of the North Denmark region, women with a pregnancy complicated by GDM are routinely informed by a nurse or midwife about the increased risk of type 2 diabetes mellitus and the recommendation of follow-up screening after birth. If the woman has insulin-treated GDM, an appointment for the first screening is scheduled at two to three months after birth, while it is left to women with noninsulin-treated GDM to book a screening appointment with their GP. In all cases, the woman is responsible for booking further screening appointments in the years following birth in general practice. Overall, the region has high participation rates for the first screening, while a significant decline is observed in the following years [6]. The described study intervention consisted of an electronic reminder of screening sent to all women in the intervention group, who additionally received standard care. The email informed the women of the increased risk of type 2 diabetes and the benefits of early diabetes detection. Recommendations on screening, details on the booking procedure and a contact address for further information were given. The approach was based on the principles of informed choice, patient-centred care, and the belief that brief decision support interventions are helpful for pregnant women [18].

Women who had participated in screening in the previous 12 months were asked to disregard the reminder, which was sent through a secure nation-wide email system accessed by almost all citizens in Denmark for information from public authorities (e.g., the healthcare system, tax authorities, etc.). Women can access the Danish secured email systems by use of a mobile application. The system was thus easily accessible [19]. Citizens can apply for exception from use of this secured email system due to e.g., mental or physical illness; in the North Denmark region exception is estimated to be granted to approximately seven percent of the population. As the secure email was linked to the women’s CPR number, no further contact information was needed. The reminder was despatched on 27 August 2020.

To support adoption [8, 20] of the follow-up screening and local anchoring in the region, its GPs were informed of the project through official and organizational channels. The total cost of the entire intervention, which is likely to decrease if routinely implemented, did not exceed €1000 (secure email fee).

### Randomization

The study population was stratified on the calendar year for the GDM pregnancy, based on the birth year of the child. An independent statistician was tasked with randomization into either intervention group or control group within each stratum using R Core Team software (2020) [21] with a computer-generated random allocation sequence to create a 1:1 ratio. Determined by formal chance processes, the assignment to the intervention could not be predicted or influenced. The outcome assessor was furthermore blinded to treatment allocations, which prevented bias in the estimated effect of the intervention. While the nature of the intervention precluded blinding of participants within intervention groups, the control group members were unaware of the intervention study.

### Outcome assessment

The primary outcome was any participation in follow-up screening after receiving a reminder, defined as the performance of the recommended blood test for diabetes. In general practice, HbA1c are the recommended diagnostic test used for women with previous GDM [13]. The HbA1c measurement (mmol/mol) complies to the more recent standardization from the International Federation of Clinical Chemistry (IFCC) [13]. The tests performed in the general practice clinics could however be either an oral glucose tolerance test (OGTT) or a fasting P-glucose test, and blood samples for HbA1c which were sent from GPs for analysis in a regional biochemical department. Different data sources were therefore combined to assess the primary outcome. The registry of National Health Insurance Statistics contains information on health insurance services made in general practice [14], and pay-per-performance principles ensured registration and monitoring of the screening tests. The performance code for GP clinics’ testing of blood glucose was used (Code: 7136). GPs could also request a P-glucose analysis from hospitals’ biochemical departments, which used NPU (Nomenclature for Properties and Units) terminology, an international coding system that enables identification of test results [22]. NPU codes used to determine performed test are NPU27412 (HbA1c), NPU21530 (OGTT) and NPU02192 (P-glucose). The secondary outcome was a diagnosis of type 2 diabetes mellitus defined by HbA1c test values ≥ 48 mmol/mol as identified through the NPU coding, or a diabetes diagnosis (ICD10:DE10-14) registered by the hospital. Outcome data were retrieved in February 2021, approximately 6 months after reminders were despatched.

### Data analysis

The baseline characteristics of all randomized women were compared descriptively, showing frequency and percentages in total and between the control and intervention groups. Categorizations of baseline characteristics included Age (≤ 25 Years, 26–35 Years, 36–50 Years), Ethnicity (Danish/western, non-western), Employment status in percentages (defined as number of weeks as self-supporting, including women on maternity leave and state education support 0–2 years before despatch of reminders) (≥ 80%, 20–80%, ≤ 20%) [23], Municipality (urban: 219,500 inhabitants in one municipality, rural: 371,000 inhabitants across 10 municipalities), Parity (primipara, multipara) and Body mass index kg/m^2^ (BMI; underweight/normal (BMI < 25), overweight (BMI 26–30), obese (BMI > 30).

We estimated the effect of the intervention by reporting risk ratios (RR), risk differences (RD) and 95% confidence intervals (CI) for primary outcomes. To calculate the number of women diagnosed with diabetes and a *P*-value in relation to the secondary outcome, Pearson’s chi square test was used. Outcome comparisons were made according to randomization allocations, including women who did not receive the reminder intervention as intended. This preserved the integrity of randomization during analysis and respected the intention-to-treat principle. A histogram was prepared to graphically display frequency and distribution of the primary outcome in the time after despatching the reminder to the intervention group. Distributions of the primary outcome was made according to five timeline cut-offs (August/September, October, November, December, and January/February).

A forest plot graphically displayed the estimated results according to stratified groups representing years after birth. To estimate the effect of the intervention for different subgroups, we also stratified for age, ethnicity, employment status, municipality, parity, and BMI. All statistical analyses were performed using Stata 16.1 software for Windows® (StataCorp., College Station, TX, USA).

## Results

### Recruitment and participant flow

**Fig. 1:**
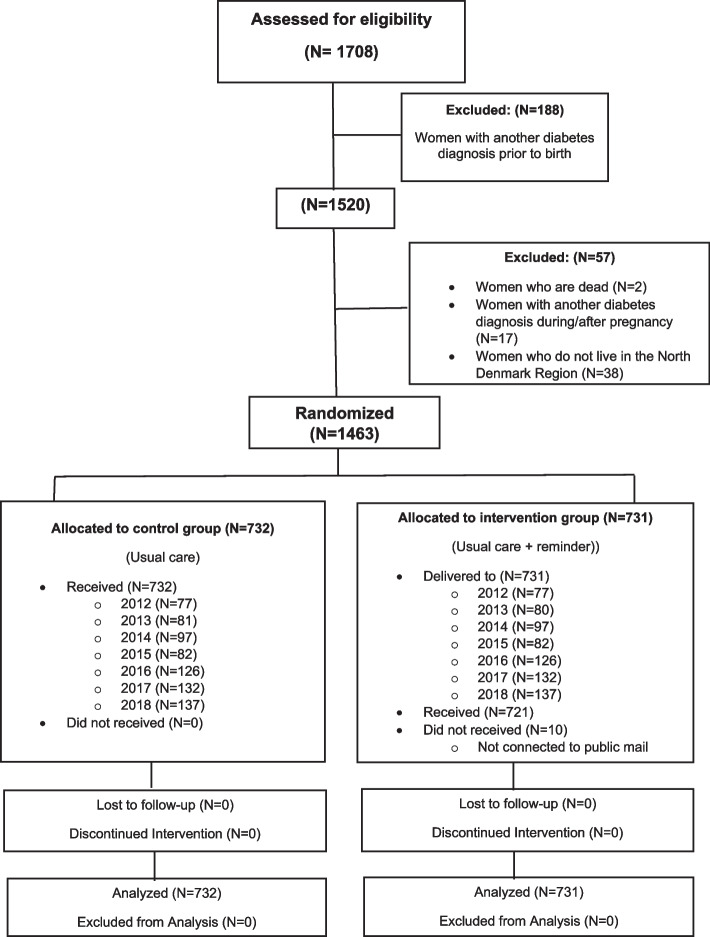
Flowchart

Of the 1708 women assessed for eligibility, 188 had a diabetes diagnosis prior to birth (Fig. 1). Additionally, of the 57 excluded women, two had died before study onset, 17 had been diagnosed with diabetes during/after pregnancy prior to the intervention, and 38 no longer lived in the North Denmark Region (Fig. 1). Participants were randomized to either control group/standard care (*n* = 732) or intervention group/standard care and an email reminder (*n* = 731). Ten (1.37%) of the 731 women in the intervention group did not use the secure email system (no reason reported). These women belonged to the following groups stratified by year: 2013 (two women), 2014 (four women), 2016 (three women) and 2017 (one woman). In accordance with the intention-to treat-principle, the ten women were included as part of follow-up leading to a complete follow-up of the 1463 women.

### Characteristics of included women

There were no notable differences between the control group and the intervention group at trial entry (Table 1). Both groups were dominated by women between 26–35 years of age (≈ 66%), of Danish or western decent (≈ 88%). With mostly self-supporting women, and approximately 13% receiving public benefits for more than 80% of the time, the included group of women reflected the general socio-economic conditions of women at childbearing age. Equal numbers of women lived in urban municipalities and rural municipalities; the same applied to parity. As could be expected for women with prior GDM, many (≈ 41%) had a BMI > 30 before pregnancy and were categorized as obese.

**Table 1:** Baseline characteristics of women included in study

Factor	Total (*n* = 1463) (%)	Intervention (*n* = 731) (%)	Control (*n* = 732) (%)
**Age:**			
≤ 25 Years	118 (8.0)	63 (8.6)	55 (7.5)
26–35 Years	958 (65.5)	466 (63.7)	492 (67.4)
36–50 Years	385 (26.3)	202 (27.6)	183 (25.0)
Missing	2 (0.1)	0 (0.0)	2 (0.2)
**Ethnicity:**			
Danish/western	1253 (88.1)	628 (88.8)	625 (87.5)
Non-western	168 (11.8)	79 (11.1)	89 (12.4)
Missing	42 (2.8)	24 (3.2)	18 (2.5)
**Employment status**^a^**:**			
≥ 80%	1008 (68.9)	501 (68.6)	507 (69.2)
20–80%	273 (18.6)	141 (19.2)	132 (18.0)
≤ 20%	182 (12.4)	89 (12.1)	93 (12.7)
**Municipality**^b^**:**			
Urban	691 (47.2)	335 (45.8)	356 (48.6)
Rural	772 (52.7)	396 (54.1)	376 (51.3)
**Parity:**			
Primipara	668 (45.6)	335 (45.8)	333 (45.4)
Multipara	794 (54.3)	395 (54.1)	399 (54.5)
Missing	1 (0.07)	1 (0.1)	0 (0.0)
**BMI kg/m**^**2**c^**:**			
< 25	418 (28.7)	211 (28.9)	207 (28.5)
25–30	437 (30.0)	218 (29.9)	219 (30.1)
> 30	600 (41.2)	300 (41.1)	300 (41.3)
Missing	8 (0.5)	2 (0.2)	6 (0.8)

^a^Defined as number of weeks as self-supporting, including on maternity leave or receiving education grant 0–2 years before despatch of reminder, calculated in percentage (≥ 80%, 20–80%, ≤ 20%)

^b^Urban (219,500 inhabitants in 1 municipality), rural (371,000 inhabitants across 10 municipalities)

^c^*BMI* Body mass index kg/m^2^: Underweight/normal (BMI < 25), overweight (BMI 25–30), obese (BMI > 30)

### Postpartum follow-up and early detection of diabetes

Screening involved 471 (32.1%) women. The primary outcome event was experienced by 257 women (35.1%) in the intervention group and 214 women (29.2%) in the control group. We demonstrated a 20% increased chance of participation in screening in the intervention group (RR: 1.20; 95% CI 1.03–1.39) and a 5% increase in absolute risk (RD: 0.05; 95% CI 0.01–0.10] (Table 2).

**Table 2:** Outcome: participation in screening

Outcome	Total (*n* = 1463) (%)	Intervention (*n* = 731) (%)	Control (*n* = 732) (%)	Relative risk (95% CI)	Risk difference
Women participating in screening	471 (32.1)	257 (35.1)	214 (29.2)	1.20 (1.03–1.39)	0.05 (0.01–0.10)

Screening was performed using a HbA1c test in 415 women (NPU27412), a OGTT in 34 women (NPU21530), by P-glucose testing (NPU02192) of 14 women, while 8 women were tested according to the performance code for GPs (Code:7136).

**Fig. 2:**
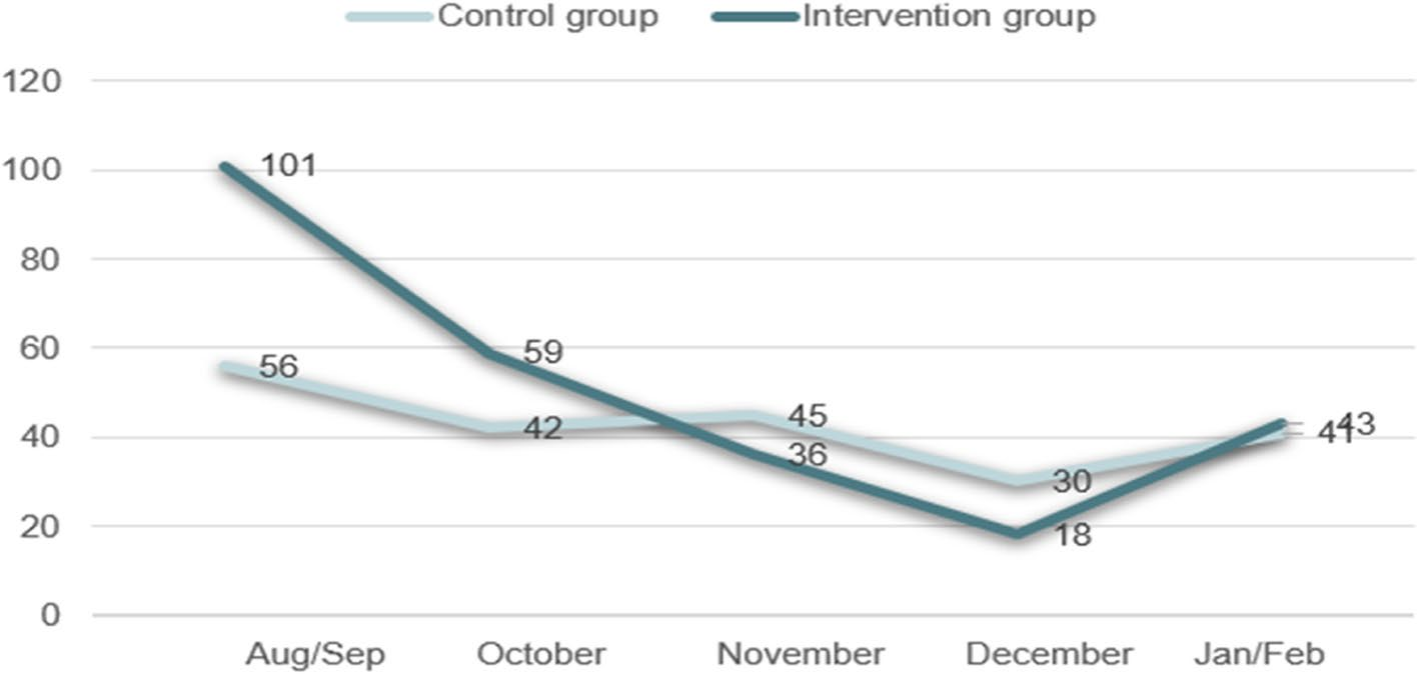
Histogram illustrating the distribution of participation in screening in time after despatching the reminder

The effect was highest immediately after the reminder was sent out in August/September and October (Fig. 2).

**Fig. 3:**
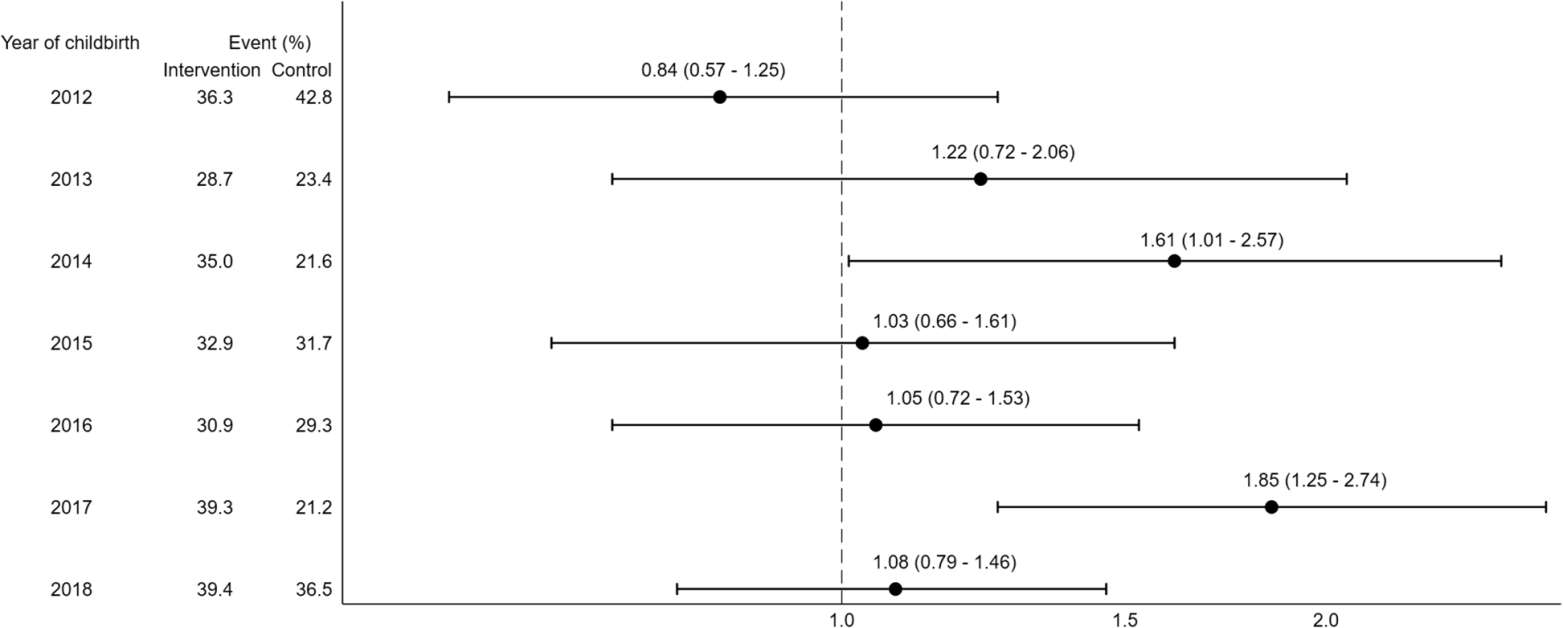
Forest plot illustrating the effect of the reminder according to stratified groups representing years after birth

Figure 3 shows that in all years, except 2012, the proportion of women who participated in screening were highest in the intervention group.

Among the women who participated in follow-up screening, 56 (3.8%) were diagnosed with type 2 diabetes mellitus after the reminder was despatched.

The secondary outcome (type 2 diabetes diagnosis) was detected in 32 women within the intervention group and in 24 women in the control group. No significant difference was found (*P*-value: 0.27). Most of the women with type 2 diabetes mellitus were diagnosed according to HbA1c > 48 mmol/mol (54 women); only two women were identified through hospital ICD-10 codes.

### Subgroup analyses

Compared with women receiving standard care, women in the intervention group showed significantly higher participation rates if they were of Danish or western decent, 26–35 years old, multipara, lived in an urban area and with a BMI < 25 (Table 3). The risk difference, i.e., the difference in the probability of experiencing the event, appeared to increase with age and with non-western origin, urban dwelling, multiparity, and for women with BMI < 25.

**Table 3:** Subgroup analysis

Subgroups	Total (*n* = 1463) (%)	Intervention (*n* = 731) (%)	Control (*n* = 732) (%)	Relative risk (95% CI)	Risk Differenceis
**Age:**					
≤ 25 Years	28 (23.7)	15 (23.8)	13 (23.6)	1.00 (0.52–1.92)	0.00 (-0.15–0.16)
26–35 Years	321 (33.5)	171 (36.7)	150 (30.4)	1.20 (1.00–1.43)	0.06 (0.00–0.12)
36–50 Years	122 (31.6)	71 (35.1)	51 (27.8)	1.26 (0.93–1.70)	0.07 (-0.01–0.16)
**Ethnicity:**					
Danish/western	409 (32.6)	222 (35.3)	187 (29.9)	1.18 (1.00–1.38)	0.05 (0.00–0.10)
Non-western	57 (33.9)	31 (39.2)	26 (29.2)	1.34 (0.87–2.05)	0.10 (-0.04–0.24)
**Employment status**^a^**:**					
≥ 80%	320 (31.7)	172 (34.3)	148 (29.1)	1.17 (0.98–1.41)	0.05 (-0.00–0.10)
20–80%	83 (30.4)	49 (34.7)	34 (25.7)	1.34 (0.93–1.94)	0.08 (-0.01–0.19)
≤ 20%	68 (37.3)	36 (40.4)	32 (34.4)	1.17 (0.80–1.71)	0.06 (-0.08–0.20)
**Municipality**^b^**:**					
Urban	216 (31.2)	124 (37.0)	92 (25.8)	1.43 (1.14–1.79)	0.11 (0.04–0.18)
Rural	255 (33.0)	133 (33.5)	122 (32.4)	1.03 (0.84–1.26)	0.01 (-0.05–0.07)
**Para:**					
Primipara	233 (34.8)	121 (36.1)	112 (33.6)	1.07 (0.87–1.32)	0.02 (-0.04–0.09)
Multipara	238 (29.9)	136 (34.4)	102 (25.5)	1.34 (1.08–1.67)	0.08 (0.02–0.15)
**BMI kg/m**^**2** c^**:**					
< 25	123 (29.4)	76 (36.0)	47 (22.7)	1.58 (1.16–2.16)	0.13 (0.04–0.21)
25–30	135 (30.8)	68 (31.1)	67 (30.5)	1.01 (0.77–1.34)	0.00 (-0.08–0.09)
> 30	209 (34.8)	113 (37.6)	96 (32.0)	1.17 (0.94–1.46)	0.05 (-0.01–0.13)

^a^Defined as number of weeks as self-supporting or receiving public benefits 0–2 years before despatch of reminder, calculated in percentages

^b^Urban (219,500 inhabitants in 1 municipality), rural (371,000 inhabitants across 10 municipalities)

^c^BMI, body mass index kg/m^2^: Underweight/normal (BMI < 25), overweight (BMI 25–30), obese (BMI > 30)

### Identified harms

No significant harms were identified as a result of the study.

## Discussion

### Key results

This study demonstrates a significant increase in participation in screening among women with prior GDM who receive an email reminder in comparison with women who receive only standard care. Our results thereby corroborate the growing body of evidence that reminding women to be screened after birth are effective [9, 24, 25]. Our study, is to the best of our knowledge the first to test the effect of a reminder beyond the first 12 months after birth [8], and the studied intervention appears to support the recommendation of participation in screening [5]. As the women in our study received the reminder only once, we have no knowledge of the potential of yearly reminders. Reminder fatigue can occur over time with increasing numbers of reminders but is not inevitable [26]. Since the majority of guidelines recommend recurrent annual follow-up screenings, or at least every three years after birth, the use of annual reminders may increase adherence even further. Subgroup analysis has moreover suggested that women of non-western origin are more likely to respond to the reminder. This is interesting, as previous published literature suggest that women of non-Western can be hard to engage in screening [27]. Also, our analysis suggests that reminders are supportive for multiparas, however even when adding a reminder to usual care participation of multiparas do not quit get on the same level of participation as primiparas. This could indicate that it can be hard to prioritize screening when joggling family related practicalities, something which have been identified in previous studies [27].

Contextual factors may explain the variations in effect found between this study and previous studies [8, 9]. Unlike the situation in many other countries, our Danish study setting provided universal and free access to healthcare and life-long follow-up screening. As pointed out in a published RCT study, this seems to be central to the effect of reminders, as participation was significantly higher in patients covered by public healthcare [10].

Our study also found a stronger effect of the reminder among urban women. However, it is important to notice that women from rural areas in general seems to participate in screening more than women from urban areas but might not be especially responsive to a reminder.

The reminder nevertheless seems to support continuity of care for women with previous GDM, a group that has expressed discontent about fragmented care and little opportunity to receive elaboration on health risks and recommendations [28]. This problem is documented by international research, which found that women experience care as particularly fragmented on their return to general practice care following a hospital birth [27].

Drawing on the unique possibility offered by Denmark’s civil registration number system to link individual data across multiple nationwide registers, our simple intervention design enabled us to identify and recruit participants, despatch the reminders, and assess outcomes without causing any significant disturbances for current practices. This ensured sufficient recruitment and retention rates with no loss to follow-up, a frequent challenge to the feasibility of executing interventions studies [29]. In comparison to similar interventions studies, our sample was sizable and retention satisfactory [24, 25, 30–32]. The use of an existing secure email system involving almost all Danish citizens also helped ensure the reminders near total delivery rate. Only about 1% of the intervention group population failed to receive the reminder, which is an improvement on similar intervention studies [16, 17]. Neither did local changes or modifications during implementation and delivery alter the intervention’s content, which may otherwise challenge the fidelity of more complex intervention designs [29]. The low intervention costs also support the feasibility and sustainability of similar interventions in the future. However, as the highest effect of the reminder is found briefly after despatch, we recommend that revisions of local guidelines benefit from discussions of frequency and time of despatching reminders. Finally, the recommended test for screening in Denmark (HbA1c) could have eradicated previous barriers, such as the discomfort of the OGTT or that fasting was needed [27].

Reflection on coverage of an intervention in a specific service setting gives indications of its integration [29]. Our study’s overall participation rate of 32.1% may be considered low in comparison with other studies, whose rates range from 44 to 76% in the first 12 months after birth [16, 17, 24]. However, the long-term perspective of this study allows us to disclose significantly higher participation rates in the later follow-up screenings compared with those previously found in the region (approximately 17% of women participating in screening 4–6 years after birth) [6]. This may suggest that decision aids such as electronic reminders can be effective in enhancing informed decision-making about participation in screening programmes [18], as recommended by the World Health Organization that high uptake in screening should not take precedence over women’s informed choice concerning participation in screening according to their individual values and preferences [33].

However, as poor communication across sectors and GP clinics’ insufficient information on risks and recommendations may challenge participation in follow-up screening [27, 28], initiatives to further increase the effect of reminders should be prioritized. Attempts to increase attention to screening stimulate the participation of women burdened by everyday life obligations [27], including those who have expressed a wish to attend follow-up screening [28]. Reminders targeting both physicians and women [34] in combination with staff training and other initiatives [16, 25], have been established as effective and should be considered in the effort to overcome barriers. However, the implementation of reminders appears to pose a challenge to general practice clinics [17]. Even though the secured email systems used in this study have the above-mentioned benefits and were considered easily accessible to women in the context of Denmark, other types of reminders to women have also been shown effective in increasing participation [8, 9]. Our data also suggest that the reminder was less effectual among younger women and women with overweight. The young women’s poor response could be explained by the low-risk perception established in a survey study that found great divergence between young persons’ recognition of GDM as a general risk factor for diabetes and their assessment of own risk of diabetes [35]. Our results support previous findings that women with overweight are less likely to participate in screening due to apprehensions about receiving a type 2 diabetes mellitus diagnosis [8]. Ways to support these two groups of women should be identified.

### Strengths and limitations

The simple study design, with its adaptation to existing system resources, ensured the inclusion of practically all cases and high follow-up rates. The validity of the Danish National Patient Register database enabled the identification of women with a GDM diagnosis, lending strong support to our expectation that women receiving this diagnosis through ICD-10 coding during pregnancy/childbirth are correct [12]. We moreover attempted to ensure that misdiagnoses were excluded. The use of an RCT design for evaluating interventions is generally considered a strength as it helps prevent selection bias and potential confounding, and our baseline info ensured successful randomization.

To ensure the long-term sustainability of the reminder intervention, we included women who had been screened in the previous 12 months. Any negative consequences of receiving an irrelevant reminder were limited by encouraging the women to disregard it, but more knowledge of women’s perspectives on receiving the reminder is, however, needed to ensure that no significant harms were associated with this study. The results of a qualitative study of this are forthcoming.

In the assessment of the primary outcome, several data sources were used to secure identification of an event which according to recommendations could include three different blood tests. However, in relation to the secondary outcome, ICD-10 coding fails to securely identify those who are merely diagnosed and treated for type 2 diabetes mellitus in general practice [12]. Our additional identification of type 2 diabetes mellitus diagnosis through NPU coding nonetheless ensured satisfactory identification, hence HbA1c test was found to be the most frequently used test for screening in general practice clinics. The single-blinded design prevented bias in estimating the effect of the intervention by ensuring that the outcome assessor and the control group were blinded to treatment allocation. Moreover, the application of intention-to-treat principles in the data analysis reflects real-life practice and minimizes the chance of overestimating effects.

Our subgroup analysis contributes to the discussion of the design of future electronic reminder systems, but its results should be interpreted with caution as the numbers were small and randomization may not have been maintained in the subgroups. Moreover, knowledge of insulin therapy rates during pregnancy with GDM should be classified, as it can affect women's motivation to participate in screening. Nevertheless, the RCT design is expected to have distributed the number of women who have received insulin therapy during pregnancy equally between the control and the intervention group.

Informing GPs about the study before despatching the reminder may have increased uptake of screening in the control group, which may have resulted in an underestimation of the effect of the reminder. However, we believe this would have had a minor effect as GPs properly are inundated by information, and that the reminder was addressed to women.

### Implications for practice and research

We urge general practice clinics to continue to strengthen attempts to engage in the decision-making process with women and support knowledge transfer between healthcare sectors. Such challenges appear to have diminished the effect of the reminder, creating a barrier to follow-up screening. Even if the reminder were routinely despatched, women should be offered support for their decision-making, especially if their GDM pregnancy occurred several years earlier. Nevertheless, routine use of reminders should be considered, in order to strengthen women's opportunity to be tested and, in dialogue with general practitioners, gain information on how diabetes conversion can be prevented. It is especially important in a Danish setting where evidence-based lifestyle interventions are not systematically available to women with impaired glucose tolerance (IGT). Our work has implications for all research concerning the coverage of reminder interventions. An adjunct process evaluation to our study, which is currently being analysed, can help generate more knowledge about women's experiences of receiving the reminder and participating in screening. Also, no previous cost-effectiveness studies on the use of reminder systems to increase uptake in screening after birth for this specific group of women have been identified. Finally, should potentials of yearly reminders and more knowledge about the specific subgroups be analysed.

## Conclusion

Electronic reminders to women can support the recommendation of participation in follow-up screening, as this study test found that a reminder beyond the first 12 months after birth are effective in increasing women’s participation in screening. The reminder is based on the principles of informed choice and patient-centred care, which are believed to be a strength. The advantage of the intervention stems from its simplicity and the use of a nationwide secure email system linked to women’s CPR number, for which policy makers should analyse contextual conditions for implementation. Attempts to further stimulation of coverage could however be considered, possibly focusing on strengthen engagement of women in the decision-making process and support of knowledge transfer between healthcare sectors.

## Data Availability

All data generated or analysed during this study are included in this published article.

## Abbreviations

GDM: Gestational Diabetes Mellitus
OGTT: Oral glucose tolerance test
IDF: International Diabetes Federation
WHO: World Health Organisation
CPR: The Central Person Register number
